# Quantification of contrast agent uptake in the hepatobiliary phase helps to differentiate hepatocellular carcinoma grade

**DOI:** 10.1038/s41598-021-02499-2

**Published:** 2021-11-26

**Authors:** Michael Haimerl, Kirsten Utpatel, Andrea Götz, Florian Zeman, Claudia Fellner, Dominik Nickel, Lukas Luerken, Frank Brennfleck, Christian Stroszczynski, Alexander Scheiter, Niklas Verloh

**Affiliations:** 1 Department of Radiology, University Hospital Regensburg, 93042 Regensburg, Germany; 2 Institute of Pathology, University Hospital Regensburg, Regensburg, Germany; 3 Center for Clinical Trials, University Hospital Regensburg, Regensburg, Germany; 4 MR Application Predevelopment, Siemens Healthcare, Erlangen, Germany; 5 Department of Surgery, University Hospital Regensburg, Regensburg, Germany; 6 Department of Diagnostic and Interventional Radiology, Medical Center University of Freiburg, Freiburg, Germany

**Keywords:** Cancer imaging, Tumour immunology, Hepatocellular carcinoma, Liver, Imaging techniques, Translational research

## Abstract

This study aimed to assess the degree of differentiation of hepatocellular carcinoma (HCC) using Gd-EOB-DTPA-assisted magnetic resonance imaging (MRI) with T1 relaxometry. Thirty-three solitary HCC lesions were included in this retrospective study. This study's inclusion criteria were preoperative Gd-EOB-DTPA-assisted MRI of the liver and a histopathological evaluation after hepatic tumor resection. T1 maps of the liver were evaluated to determine the T1 relaxation time and reduction rate between the native phase and hepatobiliary phase (HBP) in liver lesions. These findings were correlated with the histopathologically determined degree of HCC differentiation (G1, well-differentiated; G2, moderately differentiated; G3, poorly differentiated). There was no significant difference between well-differentiated (950.2 ± 140.2 ms) and moderately/poorly differentiated (1009.4 ± 202.0 ms) HCCs in the native T1 maps. After contrast medium administration, a significant difference (p ≤ 0.001) in the mean T1 relaxation time in the HBP was found between well-differentiated (555.4 ± 140.2 ms) and moderately/poorly differentiated (750.9 ± 146.4 ms) HCCs. For well-differentiated HCCs, the reduction rate in the T1 time was significantly higher at 0.40 ± 0.15 than for moderately/poorly differentiated HCCs (0.25 ± 0.07; p = 0.006). In conclusion this study suggests that the uptake of Gd-EOB-DTPA in HCCs is correlated with tumor grade. Thus, Gd-EOB-DTPA-assisted T1 relaxometry can help to further differentiation of HCC.

## Introduction

Hepatocellular carcinoma (HCC) is the fifth most common malignant tumor in man and the ninth most common malignant tumor with a rising incidence and mortality^1^. HCCs arise from hepatocytes and, in most cases, in the presence of chronic liver diseases such as cirrhosis, chronic viral hepatitis, or steatohepatitis^2^. Patients at risk are strongly advised to undergo a routine liver examination twice a year via abdominal ultrasound for the early detection of malignant liver lesions^3^. Over the past few years, there has been much progress in the diagnosis of HCC, particularly in imaging techniques. The diagnostic approach can be performed either with invasive procedures, i.e., a needle biopsy of the lesions, or noninvasive procedures, such as imaging and tumor markers^4^. Invasive techniques such as biopsy show a broad range of complications, e.g., bleeding or seeding tumor cells, and have a low negative predictive value (13–75%), resulting in false-negative results. Therefore, a negative result should be clarified by either a second biopsy or imaging^5^. Alpha-fetoprotein (AFP), as a tumor marker for HCC, is an inadequate screening marker in general^6^; however, substantially increased AFP levels in patients with liver cirrhosis have a high positive predictive value for HCC^7^. According to current guidelines, lesions exceeding a diameter of 2 cm can be diagnosed as HCCs based on typical imaging criteria, such as hypervascularity in the hepatic arterial phase and washout in a later phase on dynamic contrast-enhanced computed tomography (CT) or magnetic resonance imaging (MRI)^8^. A biopsy is indicated only for smaller lesions or if the lesion's vascular profile is unclear^9^. Several studies have shown that contrast-enhanced MRI is more sensitive to the detection of HCC than dynamic contrast-enhanced CT, probably because of the better delineation of contrast between the lesion and liver and a more subtle presentation of different tissue properties^10–12^. Currently, MRI of the liver with the liver-specific contrast agent Gd-EOB-DTPA has become indispensable in the evaluation and treatment planning of malignant liver lesions.

**Figure 1:**
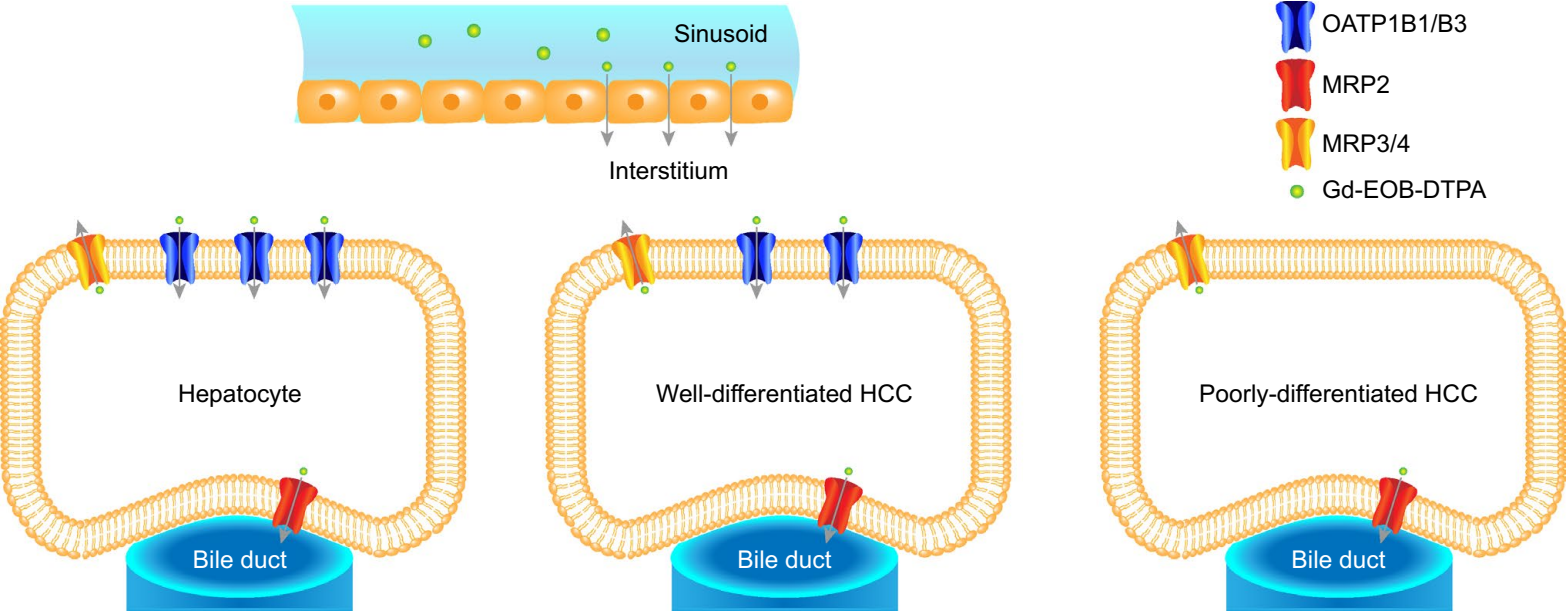
Gd-EOB-DTPA uptake. In healthy hepatocytes, the hepatobiliary contrast agent Gd-EOB-DTPA is distributed from sinusoidal spaces into the interstitium and consecutively into hepatocytes. Gd-EOB-DTPA sinusoidal clearance into the liver is controlled by the transport of organic anion-transporting polypeptides (OATPs). Absorbed Gd-EOB-DTPA passes from hepatocytes into the bile canaliculi and partially returns to the interstitium with the help of multiple resistance-associated proteins (MRP1); this intercompartmental transfer generates a ratio between the sinusoids, interstitium, hepatocytes, and bile canaliculi. In tumor cells, the expression of MRP is often maintained, whereas the expression of OATP1B1/B3 tends to decrease over the course of hepatocarcinogenesis^14^.

The uptake of Gd-EOB-DTPA, a hepatocyte-specific contrast agent, depends on functioning hepatocytes via organic anion transporters (OATPB1/B3), and it is excreted through the biliary tract via multidrug resistance-associated protein 2 (MRP2, Fig. 1)^13,14^. With an increase in the degree of malignancy, OATP1B1 or -B3 expression tends to decrease^15^, and MRP2 expression tends to be consistent or increase in HCCs^16^, which therefore accumulate less Gd-EOB-DTPA than normal functioning liver parenchyma. As HCCs dedifferentiate over time^17^, Gd-EOB-DTPA accumulation might be related to the degree of malignancy. Since the histological grade of HCCs is also considered a relevant prognostic marker^18,19^, a preoperative assessment of the malignancy grade based on MR images may be helpful regarding the therapeutic outcomes.

A typical assessment of HCC is the qualitative evaluation of signal intensity (SI) and the relative enhancement ratios comparing Gd-EOB-DTPA uptake in liver lesions to that in the surrounding liver parenchyma. However, HCCs usually occur in cirrhotic livers^2^, which show impaired Gd-EOB-DTPA uptake^20^.

Only a few studies have been published on the evaluation of T1 mapping compared to tumor grade^21–25^. T1 mapping, in contrast to SI-based evaluations, has the possibility of a direct quantitative evaluation of liver lesion without the necessity of comparing to the surrounding tissue^21,24^. Peng et al. showed that T1 mapping before and after Gd-EOB-DTPA administration might contribute to the classification of HCC according to tumor grade with a calculated percentage reduction of T1 in HBP as the best indicator for classification^25^.

These studies have in common that the liver lesions in the T1 maps were evaluated on only one slice, resulting in a bias for lesions with regionally different uptake behavior. Therefore, in this study, we analyzed Gd-EOB-DTPA uptake in HCC lesions by calculating the change in the T1 relaxation times of the total lesion volume, using a prototypic T1 mapping technique based on the variable-flip-angle technique, which uses different flip angles to generate color-coded T1 maps. This sequence type offers a high spatial resolution and can be used for volumetric approaches.

This study aims to classify the grade of HCC malignancy by performing a quantitative volumetric assessment of the T1 reduction rate between plain and Gd-EOB-DTPA-enhanced MRI. To the best of our knowledge, no work exists that has examined the uptake behavior of Gd-EOB-DTPA in HCCs quantitatively using T1 mapping compared with immunostaining of OATP1B3, which was investigated as a secondary objective in this study.

## Results

Overall, 33 HCC lesions were analyzed in this study. 14 HCC samples were well differentiated (G1), 14 were moderately differentiated (G2), and 5 were poorly differentiated (G3). Furthermore, tumors were categorized according to the specifications of the WHO (5th edition). The mean volume of the liver lesions was 170.94 ± 212.82 cm^3^. Lesion characteristics are shown in Table 1.

**Table 1:** Tumor characteristics.

Nr	Tumor grade	Percentage of positive cells	Intensity of staining	Reduction rate	Growth pattern	HCC volume (cm^3^)	Background liver (level of fibrosis)
**High immunoreactive score (9–12)**							
1	1	0.90	+++	0.67	Conventional	196.1	Ishak 1
2	1	0.95	+++	0.59	Conventional	837.4	Ishak 0
**Medium immunoreactive score (4–8)**							
3	1	0.30	+++	0.49	Conventional	114.6	Ishak 6
4	1	0.30	+++	0.57	Conventional	118.6	Ishak 6
5	1	0.15	++	0.31	Conventional	530.1	Ishak 2
6	2	0.20	++	0.24	Conventional	282.9	Ishak 6
7	1	0.20	++	0.47	Conventional	184.3	Ishak 4
8	2	0.50	++	0.37	Conventional	135.3	Ishak 0
9	1	0.40	++	0.53	Conventional	168.9	Ishak 5
**Low immunoreactive score (1–3)**							
10	1	0.05	+++	0.40	Conventional	9.1	Ishak 3
11	2	0.03	+++	0.32	Conventional	66.8	Ishak 6
12	1	0.03	++	0.17	Conventional	11.4	Ishak 6
13	2	0.02	++	0.29	Conventional	6.1	Ishak 2
14	2	0.01	++	0.12	Conventional	285.8	Ishak 6
15	2	0.05	+	0.22	Conventional	47.1	Ishak 6
16	1	0.05	+	0.30	Conventional	0.2	Ishak 0
17	1	0.03	+	0.29	Conventional	442.9	Ishak 0
**Negative immunoreactive score (0)**							
18	3	0	0	0.32	Macrotrabecular	20.7	Ishak 4
19	2	0	0	0.19	Conventional	55.1	Ishak 4
20	3	0	0	0.23	Conventional	682.3	Ishak 2
21	2	0	0	0.36	Scirrhous	24.3	Ishak 1
22	1	0	0	0.21	Steatohepatitic	24.1	Ishak 6
23	1	0	0	0.36	Steatohepatitic	4.9	Ishak 6
24	1	0	0	0.31	Conventional	560.8	Ishak 1
25	3	0	0	0.31	Conventional	10.3	Ishak 3
26	2	0	0	0.31	Conventional	71.5	Ishak 6
27	2	0	0	0.15	Macrotrabecular	127.1	Ishak 6
28	2	0	0	0.23	Conventional	17.1	Ishak 0
29	3	0	0	0.15	Conventional	274.9	Ishak 6
30	3	0	0	0.23	Conventional	108.8	Ishak 5
31	2	0	0	0.29	Conventional	25.9	Ishak 1
32	2	0	0	0.22	Conventional	40.4	Ishak 1
33	2	0	0	0.26	Conventional	155.4	Ishak 1

A twofold comparison of the growth pattern and immunostaining results of OATP1B3 showed that poorly differentiated HCCs had no positive cells upon immunostaining, and the immunoreactive score (IRS) was negative in all cases. There was a trend toward a higher IRS for OATP1B3 with the increasing degree of differentiation (G1, G2). Moreover, 8 of 14 moderately differentiated HCCs (57.1%) had no positive staining; in contrast, only 3 of 14 well-differentiated HCCs (21.4%) had negative staining. Overall, only two HCCs (well-differentiated) had a high IRS for OATP1B3. Table 2 shows the relationships between growth patterns and immunostaining results.

**Table 2:** Relationship between the grading and immunostaining results.

	G1	G2	G3
**Percentage of positive cells, n (%)**			
0%	3 (9.1)	8 (24.2)	5 (15.2)
1–10%	4 (12.1)	4 (12.1)	–
11–50%	5 (15.2)	2 (6.1)	–
51–80%	–	–	–
81–100%	2 (6.1)	–	–
**Intensity of staining, n (%)**			
Absent	3 (9.1)	8 (24.2)	5 (15.2)
Weak	2 (6.1)	1 (3.0)	–
Moderate	4 (12.1)	4 (12.1)	–
Strong	5 (15.2)	1 (3.0)	–
**Immunoreactive score, n (%)**			
Negative (0)	3 (9.1)	8 (24.2)	5 (15.2)
Low (1–3)	4 (12.1)	4 (12.1)	–
Medium (4–8)	5 (15.2)	2 (6.1)	–
High (9–12)	2 (6.1)	–	–

**Figure 2:**
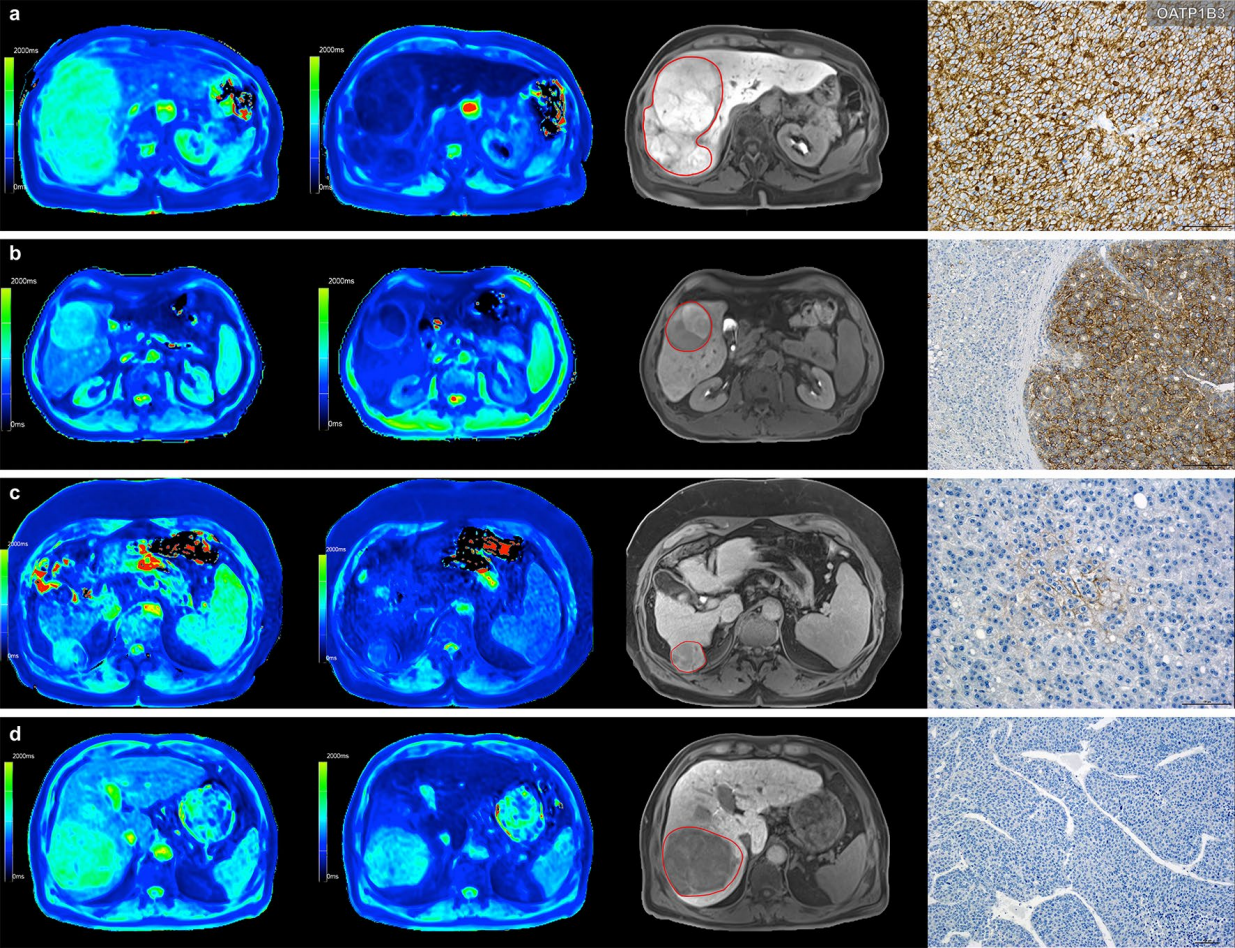
Comparison of T1 maps acquired with a variable-flip-angle MR sequence before (1st column) and 20 min after (2nd column) the administration of Gd-EOB-DTPA for HCCs (A-D) with differential OATP1B3 expression (4th column, immunostaining OATP1B3). The 3rd column displays the corresponding T1-weighted VIBE sequence with fat suppression in the hepatobiliary phase. All images displayed were taken with the same window and center level. The scale on the histopathology images represents 500 μm. (**a**) Well-differentiated HCC with high OATP1B3 expression on all tumor cells (score 12); the HCC and the surrounding liver parenchyma (normal liver parenchyma) showed high uptake of Gd-EOB-DTPA, with a reduction rate of 0.59 within the tumor. (**b**) Well-differentiated HCC with heterogeneous OATP1B3 expression: high OATP1B3 expression on the right side and low expression on the left side on tumor cells (score 6); the split within the tumor can be clearly visualized on the MR image; the T1 reduction rate was 0.49 for the HCC. (**c**) Well-differentiated HCC with minimal OATP1B3 expression on only a few tumor cells (score 1); heterogeneous uptake is also visible on the MR images, resulting in a tumor reduction rate of 0.22. (**d**) Poorly differentiated (macrotrabecular subtype) HCC with no OATP1B3 expression on tumor cells (score 0). By comparing the plain and enhanced images, only a few changes can be noticed, with a tumor reduction rate of 0.15.

**Figure 3:**
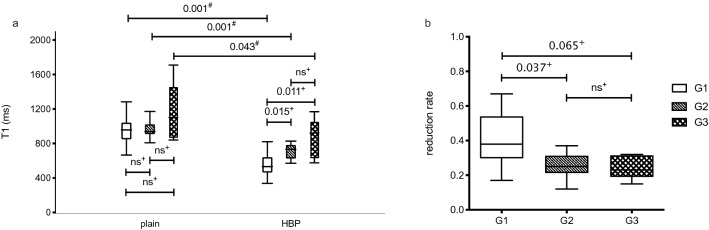
Boxplot of the T1 time and tumor reduction rate in the hepatobiliary phase. (**a**) Mean T1 time of HCC on plain and postcontrast images (HBP, hepatobiliary phase) with the relevant p-values for the comparison of Gd-EOB-DTPA uptake. (**b**) Tumor reduction rate between the plain and hepatobiliary phases for well- (G1), moderate (G2) and poorly (G3) differentiated HCCs. Data presented as box plots follow standard Tukey representations. The Wilcoxon signed rank test (#) and the post-hoc pairwise comparisons of the Kruskal–Wallis test (+) were used to compare groups.

Between plain and post-Gd-EOB-DTPA administration, there was a significant reduction in the T1 relaxation times (p = 0.001, p = 0.001, p = 0.043). There was no significant difference in the plain phase (G1, 950.2 ± 140.2 ms; G2, 959.5 ± 96.5 ms; G3 1149.3 ± 346.7 ms, p = 0.602). However, there was a significant difference in the mean T1 relaxation times for G1 lesions (555.4 ± 140.2 ms) compared to G2/G3 lesions (G2, 712.6 ± 84.4 ms, p = 0.015; G3, 858.0 ± 232.2 ms, p = 0.011) in the hepatobiliary phase 20 min after contrast administration (Fig. 2). In the pairwise comparison between G1, G2 and G3, there was a significant difference between G1 (0.40 ± 0.15) and G2 lesions (0.26 ± 0.07, p = 0.012). Comparing G1 to G3 (0.25 ± 0.07) lesions only a trend towards significancy (p = 0.065) was detected. There was no significant difference (p = 0.977) between G2 (0.26 ± 0.07) and G3 (0.25 ± 0.07) lesions (Fig. 3, Table 3). Pooling G2 and G3 into one group G2/G3, the T1 reduction rate was 0.25 ± 0.07, showing a significant difference (p = 0.006) to G1.

**Table 3:** T1 relaxation times and reduction rates for well- (G1), moderately (G2) and poorly (G3) differentiated HCCs and their surrounding liver parenchyma.

	All (n = 33)	G1 (n = 14)	G2 (n = 14)	G3 (n = 5)	p-value (Kruskal–Wallis)
**Hepatocellular carcinoma**					
T1 plain [ms]	984.3 ± 187.1	950.2 ± 140.2	959.5 ± 96.5	1149.2 ± 346.7	0.602
T1 HBP [ms]	668.0 ± 172.3	555.4 ± 140.2	712.6 ± 84.4	858.0 ± 232.2	0.003
T1 reduction rate	0.32 ± 0.13	0.40 ± 0.15	0.26 ± 0.07	0.25 ± 0.07	0.026
**Surrounding liver parenchyma**					
T1 plain [ms]	720.7 ± 158.5	738.6 ± 169.6	714.9 ± 146.5	687.0 ± 187.1	0.933
T1 HBP [ms]	332.7 ± 82.3	341.4 ± 99.8	324.1 ± 66.8	332.2 ± 92.5	0.975
T1 reduction rate	0.55 ± 0.60	0.61 ± 0.13	050 ± 0.14	0.63 ± 0.08	0.107

The values indicate the mean ± standard deviation.

**Figure 4:**
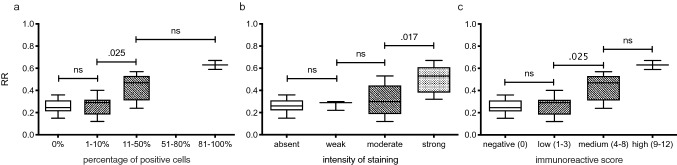
Boxplot of the tumor reduction rate in the hepatobiliary phase in comparison to immunostaining results of OATP1B3. Tumor reduction rate between the plain and hepatobiliary phases in comparison to the percentage of positive cells (**a**), the intensity of staining (**b**) and the resulting immunoreactive score (**c**, multiplication of **a** and **b**). Data presented as box plots follow standard Tukey representations. The Kruskal–Wallis test was used to compare the groups.

A comparison of the reduction rate to the IRS (Fig. 4) revealed no significant difference (p = 0.941) between a negative (median 0.25 [0.21; 0.31]) and a low IRS (median 0.29 [0.18; 0.32]). There was a significant difference (p = 0.025) between lesions with a low IRS and those with a medium IRS (median 0.47 [0.31; 0.53]). HCCs with a high IRS (median 0.63 [0.59; –) showed a higher reduction rate than HCCs with a medium IRS (p = 0.324).

We performed ROC curve analyses to differentiate G1 from G2/G3 tumors based on the tumor reduction rate. The optimal cutoff value for the tumor reduction rate to differentiate G1 from G2/G3 tumors was 0.385 (AUC 0.76). This cutoff value resulted in a sensitivity of 50% and a specificity of 100% for this differentiation, with a positive predictive value of 100% and a negative predictive value of 73%.

## Discussion

This study shows the possibility of distinguishing well-differentiated from moderately/poorly differentiated HCCs using Gd-EOB-DTPA-enhanced T1 maps. In our study, all lesions showed a reduction in the T1 relaxation time, indicating Gd-EOB-DTPA uptake. The uptake and excretion of Gd-EOB-DTPA are regulated by OATP-B1/-B3 and MRP2^13^. OATP is a multispecific transporter that is ubiquitously expressed throughout the body, with the subtypes OATP-B1 and OATP-B3 being expressed only in the liver^26^. Tsuboyama et al.^27^ proposed a hypothetical mechanism: Gd-EOB-DTPA accumulation is dependent on its uptake via OATP-B1/-B3 and its biliary excretion via MRP2. Depending on the localization of MRP2, Gd-EOB-DTPA is either secreted into canaliculi and therefore excreted into the bile, showing minor enhancement in hepatocytes, or into pseudoglands with consecutive accumulation and therefore strong enhancement.

In advanced HCCs, it has been shown that OATP-B1 or -B3 expression is usually reduced^15^ and that MRP2 expression is consistent or increased^16^; hence, these results support our findings of less Gd-EOB-DTPA uptake in G2/G3-differentiated HCCs either due to less uptake or a higher biliary excretion rate.

Not only Gd-EOB-DTPA but also a variety of substances, including atorvastatin^28^ and cefazolin^29^, and anticancer drugs, such as methotrexate^30^, rifampicin^31^, paclitaxel, and docetaxel^32^, are transported by OATP-B1 and OATP-B3. Therefore, the anticancer drug effect might be competitive and dependent on the degree of tumor differentiation correlating with the expression of these polypeptides.

Previous studies have shown that HCCs present themselves differently on Gd-EOB-DTPA-enhanced MRI depending on their grade^21,25,27,33–40^. Frericks et al.^39^ and Schelhorn et al.^34^ showed no correlation between tumor grade and the enhancement pattern; however, histopathological grading was performed only on biopsy samples instead of resected liver tissues. The evaluation was based on signal intensities compared to the surrounding liver parenchyma, and underlying cirrhosis and impaired Gd-EOB-DTPA uptake were not considered. However, Tsuboyama et al.^27^ showed OATP-B3 overexpression in all stages of differentiation, linking strong lesion enhancement to the altered expression of MRP2 due to the accumulation of Gd-EOB-DTPA. They also reported that enhancement was more pronounced than that in the liver parenchyma regardless of liver function. As they analyzed only five lesions with strong enhancement, no statement could be made about the correlation between tumor grade and strong enhancement.

For quantitative assessments, some studies, including ours, used T1 maps and measured changes in the T1 relaxation times after contrast administration to determine Gd-EOB-DTPA uptake. In the pre-interventional analysis, image-based methods with the additional T1 maps are beneficial as they allow a superior, quantitative function evaluation, compared to SI-based evaluations^41^, by maintaining morphological information simultaneously, offering a "one stop shot" approach^42^. Mio et al. showed the potential of T1 mapping for quantitative evaluation of focal liver lesions with the shortest pre- and post-contrast T1 values for HCC^24^; they concluded that T1 mapping is useful for differentiating HCC from hemangioma, metastatic tumor, and cysts, enabling accurate diagnosis.

Only a limited number of studies are available investigating the potential of plain T1 mapping to characterize tumors. Keller et al. showed the potential of plain T1 mapping as a reliable non-invasive method for quantification and characterization of primary liver tumors in a rabbit model^23^. In our study, G3 tumors had a slightly higher mean T1 time than G1 and G2 tumors. However, no significant difference was found between the different degrees of HCC differentiation in plain T1 maps (Table 3).

Huang et al.^21^ investigated several features, such as tumor size, margin, signal homogeneity, and their correlation to histological grade, showing that a larger diameter, a more irregular margin, the existence of vessels inside the lesions, and peritumoral hypointensity are signs of a lesser differentiated HCC; however, there was no correlation between the relative reduction in T1 and tumor grade. As they defined G1 and G2 lesions as low grade due to the small number of G1 lesions in their study (only eight) and compared them to G3 lesions (as medium grade), the findings are in some way consistent with those of Peng et al.^25^, who showed a correlation between G1 and G2 lesions as well as G1 and G3 lesions, but no correlation between G2 and G3 lesions. These findings are also consistent with our results. There was a significant difference in the amount of Gd-EOB-DTPA uptake between well-differentiated G1 and moderately/poorly differentiated G2 or G3 HCCs.

Presurgical determination of the degree of malignancy is essential for choosing the right therapy. According to current German guidelines, the treatment of choice for HCC in a cirrhotic liver is a liver transplant that also cures the underlying cirrhosis if the Milan criteria apply (meaning only one lesion less than five centimeters or three lesions less than 3 cm and no macrovascular invasion^43^). As these strict criteria might exclude potential patients who could benefit from a liver transplant, it has been suggested that the degree of differentiation also plays a role in liver transplant selection criteria^44,45^, as it has been shown that the histopathological grade is a prognostic factor for the outcome^19,46^.

Due to their poor prognosis, patients with poorly differentiated lesions are excluded from liver transplantation in some centers and treated noncuratively^47,48^; therefore, the importance of determining the correct degree of differentiation is evident.

The histopathological grade is currently determined by biopsy, the most common procedure being needle core biopsy, which is associated with some complications, such as bleeding, infection, and tumor seeding^49^. Although its specificity can reach up to 100%, its sensitivity can be as low as 34.6% (preoperative biopsy rather than undergraded tumors). Therefore, it has been suggested for clinicians not to rely on the histopathological grade alone for liver transplant eligibility^50^. Therefore, determining the degree of differentiation by combining radiological and histopathological features might result in greater certainty regarding the grade of HCC lesions.

This study has several limitations, including the small number of included lesions. Due to the limited group size, only five G3 HCCs were included due to their rare occurrence^51^. More than half of the lesions had to be excluded for various reasons, such as those that were pretreated or less than 1 cm in size. As a consequence of the screening program for patients at risk for HCC, more lesions are detected at an early stage^52^; therefore, they are too small to be diagnosed correctly. Due to organ shortages, HCCs are sometimes pretreated to bridge liver transplantation^53^, changing the morphology of the lesions. HCCs show high intratumor heterogeneity, which is a challenge for both pathologists and radiologists, as different degrees of differentiation might exist in one tumor^54^. This heterogeneity is seen in the high SD rates of T1 relaxation times. To reduce inaccuracies in assessing T1 relaxation times, we measured them three-dimensionally by segmentation.

Nevertheless, this study's results suggest that Gd-EOB-DTPA uptake in HCCs is correlated with the degree of malignancy. Thus, Gd-EOB-DTPA-assisted T1 relaxometry can help to further differentiation of HCC.

## Material and methods

### Study group

This study was performed as a retrospective subgroup analysis on a prospective study to evaluate contrast uptake through T1 mapping. The local institutional ethics committee of the University Hospital Regensburg approved this retrospective analysis, and the study was performed following the relevant guidelines and regulations. The ethics committee waived informed consent (Ethics Committee, University of Regensburg, 93040 Regensburg, Germany).

Between January 2014 and May 2019, 56 patients with HCC underwent Gd-EOB-DTPA-enhanced MRI of the liver, including T1 mapping. This was due to the presence of a liver lesion that was, after resection, histopathologically confirmed as HCC. Lesions less than 1 cm in size (n = 6), those with infiltrative growth (n = 1), histopathologically confirmed mixed tumors (n = 3), previously treated lesions (n = 1), lesions that went repeat MRI before resection (n = 2) or lesions with technical errors (n = 10), e.g., image artifacts in the area of interest, were excluded. Thus, a total of 33 HCC lesions were included.

### Imaging

MR images of the liver were obtained using a clinical whole-body 3 T system (MAGNETOM Skyra, Siemens Healthcare, Erlangen, Germany) with a combination of body-spine array coil elements (an 18-channel body matrix coil and a 32-channel spine matrix coil) for signal reception. Gd-EOB-DTPA (Primovist®, Bayer Vital GmbH, Leverkusen, Germany) was used as a hepatocytic contrast agent. All patients received a body weight adapted dose of Gd-EOB- DTPA (0.025 mmol/kg body weight) administered via bolus injection with a flow rate of 1 mL/s, flushed with 20 mL NaCL.

T1 maps before and 20 min after contrast injection were generated from a prototypical T1-weighted volume-interpolated breath-hold examination (VIBE) sequence (TR 5.79 ms, TE1 2.46 ms, TE2 3.69 ms) by using a prototypic technique based on a 3D spoiled-gradient echo sequence with variable flip angles (1°, 7° and 14°) and a voxel size of 3.6 mm × 2.5 mm × 4.7 mm interpolated to 1.3 mm × 1.3 mm × 3.0 mm^55,56^. In addition, a B1 map of the liver was acquired for each patient prior to the T1 relaxometry measurements to perform B1 correction to improve the homogeneity of the T1 maps^57^, and color-coded T1 maps were calculated inline. Using controlled aliasing in parallel imaging results in higher acceleration (CAIPIRINHA) as a parallel imaging technique with an acceleration factor of 4, the entire liver was covered during a single breath-hold (acquisition time 17 s).

### Image processing

Open-source Horos imaging software (Horos Project, Annapolis, MD, USA) was used to assess the volume and mean T1 relaxation time three-dimensionally by manual segmentation of the lesions in the T1 maps before and after contrast medium administration. The HCC was segmented using a closed polygon selection. The boundary of the HCC was marked in every second slice. After outlining one-half of the slices, the missing ones were calculated by interpolation. The segmentation was subsequently transported to the plain phase and manually adjusted for different respiratory levels or patient movement. The "repulsor tool" was used to manually adjust the regions of interest (ROIs) with particular care to ensure that the lesion was completely captured. The volumes were calculated by multiplying surface and slice thickness.

An additional ROI was placed manually in the surrounding liver parenchyma (with identical sizes and locations in the non-contrast and post-contrast T1 HBP), excluding visible vessels and imaging artefacts.

The uptake of Gd-EOB-DTPA was determined by calculating the reduction rate in the T1 relaxation time between plain (T1_plain_) and Gd-EOB-DTPA-enhanced (T1_HBP_) MRI as follows:

$${\text{Reduction }}\;{\text{rate}}\; {\text{in}} \;{\text{the}} \;{\text{T}}1 \;{\text{relaxation }}\;{\text{time }}\;\left( {{\text{RR}}} \right) = \frac{{{\text{T}}1_{{{\text{plain}}}} - {\text{T}}1_{{{\text{HBP}}}} }}{{{\text{T}}1_{{{\text{plain}}}} }}$$

The T1 relaxation times and T1 reduction rates were then correlated with the histopathologically determined degree of HCC differentiation.

### Histological assessment

#### Histopathological analysis

All tissue samples were obtained from routine therapeutic surgeries performed between 2014 and 2019. After fixation in neutral buffered formalin, all tissue specimens were embedded in paraffin. According to a standard protocol, 4 μm thick tissue sections were cut and stained with hematoxylin and eosin (HE). Histological and immunohistochemical analysis were performed by 2 experienced liver pathologists (A.S. and K.U.). The histological grade of HCC was determined according to the World Health Organization (WHO) criteria^51^. HCCs were subdivided into three groups according to their degree of malignancy: G1, well differentiated; G2, moderately differentiated; and G3, poorly differentiated.

#### Immunohistochemical staining of OATP1B3

Formalin-fixed and paraffin-embedded (FFPE) tissues were sectioned at a thickness of 2–3 μm on Superfrost OT and dried for approximately 30 min at 70 °C.

Immunohistochemical staining was performed on a VENTANA BenchMark ULTRA automated slide stainer (Ventana Medical Systems, Inc., a member of the Roche Group, Tucson, AZ, United States). Antigen retrieval was performed with CC1 antigen retrieval solution (Ventana; Tris–EDTA; pH 8.0) at 100 °C for 32 min. The slides were incubated with a primary antibody recognizing OATP1B3 (Sigma-Aldrich HPA 004,943, United States; dilution 1:200) for 32 min at 36 °C, followed by visualization with the OptiView DAB IHC Detection Kit (Ventana) and counterstaining with hematoxylin.

#### Evaluation of OATP1B3 immunostaining

First, all slides were screened to assess the minimum and maximum staining intensities in the tumor cells. Only membranous immunostaining on tumor cells was determined to be immunopositive. The intensity of membranous OATP1B3 immunostaining within HCC was evaluated and categorized as absent (0) (= no evidence of staining), weak (1+), moderate (2+), or strong (3+). Then, the percentage of positively stained tumor cells was grouped into five categories (0: no positive cells; 1: < 10% positive cells; 2: 10–50% positive cells; 3: 51–80% positive cells; and 4: > 80% positive cells). Subsequently, a semiquantitative score according the IRS was calculated as a product of multiplication between the positive cell proportion score (0–4) and the staining intensity score (0–3). The IRS ranged from 0 to 12 (Table 4).

**Table 4:** T1 relaxation times and reduction rates for in correlation of the Immunoreactive score (IRS).

	T1 plain [ms]	T1 HBP [ms]	T1 reduction rate
**A: Percentage of positive cells**			
0 = no positive cells	970.1 [896.1; 1098.5]	754.0 [641.6; 822.6]	0.25 [0.21; 0.31]
1 < 10% of positive cells	878.0 [723.2; 939.0]	592.9 [537.3; 702.5]	0.29 [0.18; 0.32]
2 = 10–50% of positive cells	973.6 [930.0; 1055.7]	533.0 [433.0; 740.1]	0.47 [0.31; 0.53]
3 = 51–80% of positive cells	–	–	–
4 > 80% of positive cells	1097.1 [1036.7; –]	406.3 [338.0; –]	0.63 [0.59; –]
**B: Intensity of staining**			
0 = absent	970.1 [896,1; 1098.5]	754.0 [641.6; 822.6]	0.25 [0.21; 0.31]
1 = weak	760.0 [710.9; –]	532.0 [507.9; –]	0.29 [0.22; –]
2 = moderate	954.6 [866.6; 1043.2]	666.9 [538.0; 740.2]	0.30 [0.19; 0.44]
3 = strong	957.0 [920.2; 1066.9]	474.1 [389.0; 592.6]	0.53 [0.38; 0.61]
**IRS (multiplication of A and B)**			
0 = negative	970.1 [896,1; 1098.5]	754.0 [641.6; 822.6]	0.25 [0.21; 0.31]
1–3 = low	878.0 [723.2; 939.0]	592.9 [537.2; 702.5]	0.29 [0.18; 0.32]
4–8 = medium	1000.7 [930.0; 1055.7]	533.0 [433.0; 740.1]	0.47 [0.31; 0.53]
9–12 = high	1097.1 [1036.7; –]	406.3 [338.0; –]	0.63 [0.59; –]

The values indicate the median [q1; q3].

### Statistics

Statistical analyses were performed using IBM SPSS Statistics (version 26, Chicago, IL, USA), and all data are presented as mean ± standard deviation (SD) for normal distributed data and median [q1; q3] for non-normal distributed data. The Kruskal–Wallis test followed by post-hoc pairwise comparisons was used to analyze differences between the stages of differentiation (G1, G2 and G3). For comparison between G1 and the grouped G2/G3 lesions the non-parametric Mann–Whitney test was used. The non-parametric Wilcoxon signed rank test was used for comparisons between plain and HBP phase. Receiver operating characteristic (ROC) curve analyses were performed to differentiate between G1 and G2/G3 tumors, and the optimal cutoff was estimated according to the Youden indices. The estimated areas under the curve (AUCs) with corresponding 95% confidence intervals and the sensitivity, specificity, positive predictive and negative predictive value are reported. All tests were two-sided, and values of p < 0.05 indicated a significant difference in all statistical tests.

## Supplementary Information

Supplementary Information.

## Data Availability

The data that support the findings of this study are available within the article.

## References

[1] Jemal A (2011). Global cancer statistics. CA Cancer J. Clin..

[2] El-Serag HB (2011). Hepatocellular carcinoma. N. Engl. J. Med..

[3] Bruix J, Sherman M (2005). Management of hepatocellular carcinoma. Hepatology.

[4] Greten T (2013). Diagnosis of and therapy for hepatocellular carcinoma. Z. Gastroenterol..

[5] Müllhaupt B, Durand F, Roskams T, Dutkowski P, Heim M (2011). Is tumor biopsy necessary?. Liver Transplant..

[6] Sherman M (2001). Alphafetoprotein: an obituary. J. Hepatol..

[7] Trevisani F (2001). Serum α-fetoprotein for diagnosis of hepatocellular carcinoma in patients with chronic liver disease: Influence of HBsAg and anti-HCV status. J. Hepatol..

[8] Armengol C, Sarrias MR, Sala M (2018). Hepatocellular carcinoma: Present and future. Med. Clin. (Barc).

[9] Malek NP, Schmidt S, Huber P, Manns MP, Greten TF (2014). The diagnosis and treatment of hepatocellular carcinoma. Dtsch. Arztebl. Int..

[10] Semelka RC, Martin DR, Balci C, Lance T (2001). Focal liver lesions: Comparison of dual-phase CT and multisequence multiplanar MR imaging including dynamic gadolinium enhancement. J. Magn. Reson. Imaging.

[11] Hammerstingl R (2008). Diagnostic efficacy of gadoxetic acid (Primovist)-enhanced MRI and spiral CT for a therapeutic strategy: Comparison with intraoperative and histopathologic findings in focal liver lesions. Eur. Radiol..

[12] Ichikawa T (2010). Detection and characterization of focal liver lesions: A Japanese phase III, multicenter comparison between gadoxetic acid disodium-enhanced magnetic resonance imaging and contrast-enhanced computed tomography predominantly in patients with hepatocellular carcinoma and chronic liver disease. Invest. Radiol..

[13] Leonhardt M (2010). Hepatic uptake of the magnetic resonance imaging contrast agent Gd-EOB-DTPA: Role of human organic anion transporters. Drug Metab. Dispos..

[14] Vilgrain V, Van Beers BE, Pastor CM (2016). Insights into the diagnosis of hepatocellular carcinomas with hepatobiliary MRI. J. Hepatol..

[15] Zollner G (2005). Hepatobiliary transporter expression in human hepatocellular carcinoma. Liver Int..

[16] Nies AT (2001). Expression of the multidrug resistance proteins MRP2 and MRP3 in human hepatocellular carcinoma. Int. J. Cancer.

[17] Kudo M (2009). Multistep human hepatocarcinogenesis: correlation of imaging with pathology. J. Gastroenterol..

[18] Martins-Filho SN, Paiva C, Azevedo RS, Alves VAF (2017). Histological grading of hepatocellular carcinoma: A systematic review of literature. Front. Med..

[19] Jonas S (2001). Vascular invasion and histopathologic grading determine outcome after liver transplantation for hepatocellular carcinoma in cirrhosis. Hepatology.

[20] Verloh N (2015). Liver fibrosis and Gd-EOB-DTPA-enhanced MRI: A histopathologic correlation. Sci. Rep..

[21] Huang K (2019). Imaging biomarkers for well and moderate hepatocellular carcinoma: preoperative magnetic resonance image and histopathological correlation. BMC Cancer.

[22] Chen CY, Chen J, Xia CC, Huang ZX, Song B (2017). T1 mapping combined with Gd-EOB-DTPA-enhanced magnetic resonance imaging in predicting the pathologic grading of hepatocellular carcinoma. J. Biol. Regul. Homeost. Agents.

[23] Keller S (2020). Native T1 mapping magnetic resonance imaging as a quantitative biomarker for characterization of the extracellular matrix in a rabbit hepatic cancer model. Biomedicines.

[24] Mio M (2021). Quantitative evaluation of focal liver lesions with T1 mapping using a phase-sensitive inversion recovery sequence on gadoxetic acid-enhanced MRI. Eur. J. Radiol. Open.

[25] Peng Z (2016). Gd-EOB-DTPA-enhanced magnetic resonance imaging combined with T1 mapping predicts the degree of differentiation in hepatocellular carcinoma. BMC Cancer.

[26] Abe T (1999). Identification of a novel gene family encoding human liver-specific organic anion transporter LST-1. J. Biol. Chem..

[27] Tsuboyama T (2010). Hepatocellular carcinoma: Hepatocyte-selective enhancement at gadoxetic acid–enhanced MR imaging—correlation with expression of sinusoidal and canalicular transporters and bile accumulation. Radiology.

[28] Lau YY, Huang Y, Frassetto L, Benet LZ (2007). Effect of OATP1B transporter inhibition on the pharmacokinetics of atorvastatin in healthy volunteers. Clin. Pharmacol. Ther..

[29] Nakakariya M, Shimada T, Irokawa M, Maeda T, Tamai I (2008). Identification and species similarity of OATP transporters responsible for hepatic uptake of β-lactam antibiotics. Drug Metab. Pharmacokinet..

[30] Abe T (2001). LST-2, a human liver-specific organic anion transporter, determines methotrexate sensitivity in gastrointestinal cancers. Gastroenterology.

[31] Tirona RG, Leake BF, Wolkoff AW, Kim RB (2003). Human organic anion transporting polypeptide-C (SLC21A6) is a major determinant of rifampin-mediated pregnane X receptor activation. J. Pharmacol. Exp. Ther..

[32] Smith NF (2005). Identification of OATP1B3 as a high-affinity hepatocellular transporter of paclitaxel. Cancer Biol. Ther..

[33] Huppertz A (2005). Enhancement of focal liver lesions at gadoxetic acid–enhanced MR imaging: correlation with histopathologic findings and spiral CT—initial observations. Radiology.

[34] Schelhorn J (2016). Evaluation of combined Gd-EOB-DTPA and gadobutrol magnetic resonance imaging for the prediction of hepatocellular carcinoma grading. Acta Radiol..

[35] Fujita M (1996). Contrast enhancement with GD-EOB-DTPA in MR imaging of hepatocellular carcinoma in mice: A comparison with superparamagnetic iron oxide. J. Magn. Reson. Imaging.

[36] Kim SH (2009). Gadoxetic acid–enhanced MRI versus triple-phase MDCT for the preoperative detection of hepatocellular carcinoma. Am. J. Roentgenol..

[37] Chang W-C (2014). Histological grade of hepatocellular carcinoma correlates with arterial enhancement on gadoxetic acid-enhanced and diffusion-weighted MR images. Abdom. Imaging.

[38] Choi JW (2013). Hepatocellular carcinoma: Imaging patterns on gadoxetic acid–enhanced MR images and their value as an imaging biomarker. Radiology.

[39] Frericks BB (2009). Qualitative and quantitative evaluation of hepatocellular carcinoma and cirrhotic liver enhancement using Gd-EOB-DTPA. Am. J. Roentgenol..

[40] Narita M (2009). Expression of OATP1B3 determines uptake of Gd-EOB-DTPA in hepatocellular carcinoma. J. Gastroenterol..

[41] Haimerl M (2017). Gd-EOB-DTPA-enhanced MRI for evaluation of liver function: Comparison between signal-intensity-based indices and T1 relaxometry. Sci. Rep..

[42] Geisel D, Lüdemann L, Hamm B, Denecke T (2015). Imaging-based liver function tests-past, present and future. RoFo.

[43] Mazzaferro V (1996). Liver transplantation for the treatment of small hepatocellular carcinomas in patients with cirrhosis. N. Engl. J. Med..

[44] De Carlis L (2003). Liver transplantation for hepatocellular cancer: Should the current indication criteria be changed?. Transpl. Int..

[45] Durand F, Belghiti J, Paradis V (2007). Liver transplantation for hepatocellular carcinoma: Role of biopsy. Liver Transplant..

[46] Tamura S (2001). Impact of histological grade of hepatocellular carcinoma on the outcome of liver transplantation. Arch. Surg..

[47] Cillo U (2004). Liver transplantation for the treatment of moderately or well-differentiated hepatocellular carcinoma. Ann. Surg..

[48] DuBay D (2011). Liver transplantation for advanced hepatocellular carcinoma using poor tumor differentiation on biopsy as an exclusion criterion. Ann. Surg..

[49] Takamori R, Wong LL, Dang C, Wong L (2000). Needle-tract implantation from hepatocellular cancer: Is needle biopsy of the liver always necessary?. Liver Transplant..

[50] Pawlik TM (2007). Preoperative assessment of hepatocellular carcinoma tumor grade using needle biopsy: Implications for transplant eligibility. Ann. Surg..

[51] Fletcher CD, Unni K, Mertens F (2002). World Health Organization Classification of Tumours. Pathology and Genetics of Tumours of Soft Tissue and Bone.

[52] Zhang B-H, Yang B-H, Tang Z-Y (2004). Randomized controlled trial of screening for hepatocellular carcinoma. J. Cancer Res. Clin. Oncol..

[53] Fontana RJ (2002). Percutaneous radiofrequency thermal ablation of hepatocellular carcinoma: A safe and effective bridge to liver transplantation. Liver Transplant..

[54] Kenmochi K, Sugihara S, Kojiro M (1987). Relationship of histologic grade of hepatocellular carcinoma (HCC) to tumor size, and demonstration of tumor cells of multiple different grades in single small HCC. Liver.

[55] Yoon JH, Lee JM, Kim E, Okuaki T, Han JK (2017). Quantitative liver function analysis: Volumetric T1 mapping with fast multisection B1 inhomogeneity correction in hepatocyte-specific contrast-enhanced liver MR imaging. Radiology.

[56] Deoni SC, Rutt BK, Peters TM (2003). Rapid combined T1 and T2 mapping using gradient recalled acquisition in the steady state. Magn. Reson. Med..

[57] Chung S, Kim D, Breton E, Axel L (2010). Rapid B1+ mapping using a preconditioning RF pulse with TurboFLASH readout. Magn Reson Med.

