# Supplementary material for: Quantification of contrast agent uptake in the hepatobiliary phase helps to differentiate hepatocellular carcinoma grade
Source: Sci Rep. 2021 Nov 26;11:22991. doi: 10.1038/s41598-021-02499-2 (PMC8626433; doi:10.1038/s41598-021-02499-2)
Supplement: Supplementary file 1 — Supplementary Information. [file 41598_2021_2499_MOESM1_ESM.docx]

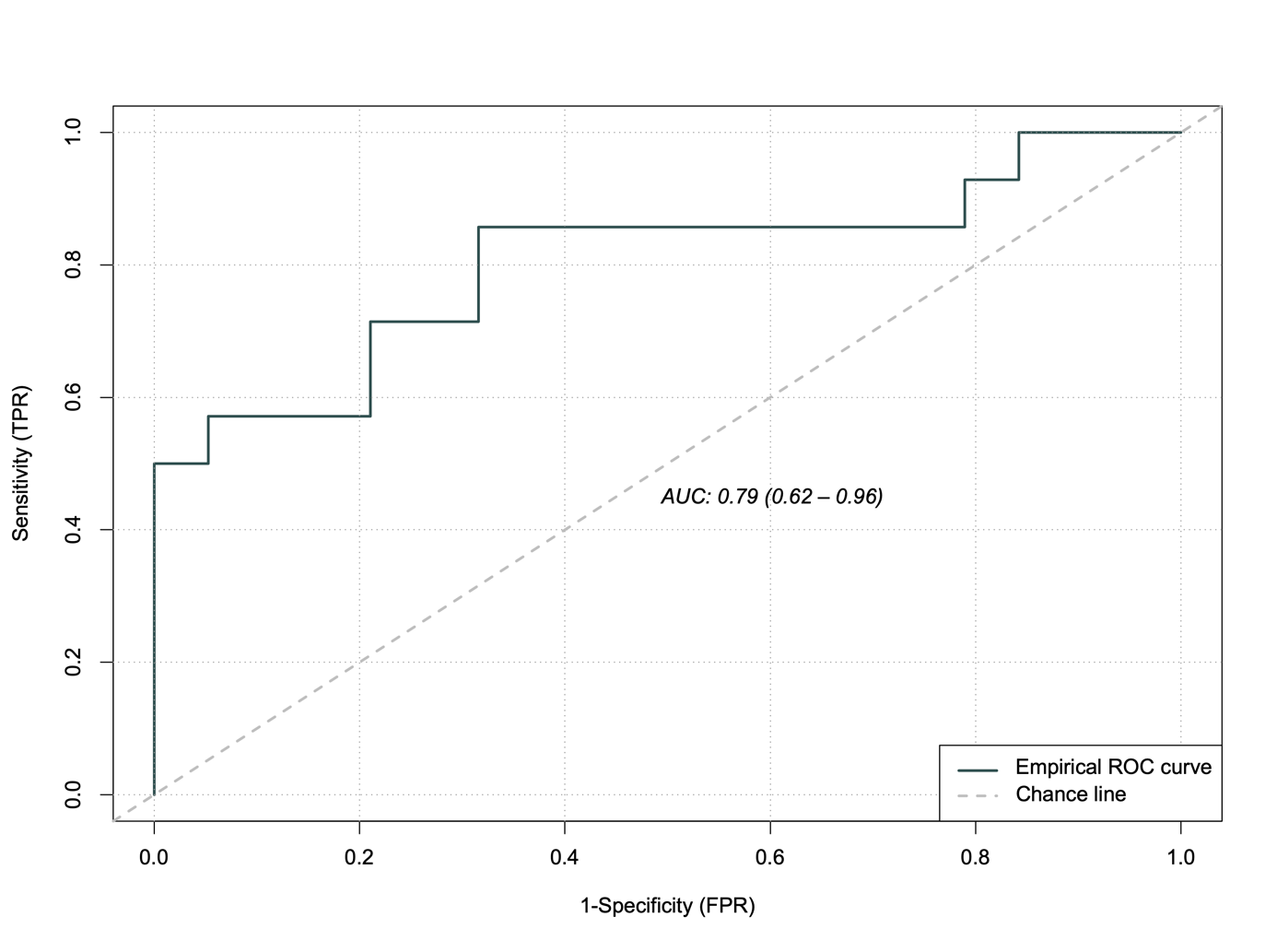


ROC Analysis

Graphs illustrating the ROC curve for the tumor reduction rate for the diagnoses of G1 in comparison to G2/G3 tumors. The optimal cut-off value for tumor reduction rate to differentiate G1 from G2/G3 tumors is 0.385 (AUC, area under the curve 0.76). This cut-off value represents a sensitivity of 50% and a specificity of 100% for this differentiation, with a positive predictive value of 100% and a negative predictive value of 73%.
